# A158 FINDINGS FROM A MULTIDISCIPLINARY DATABASE FOR PATIENTS WITH BARRETT'S ESOPHAGUS AND EARLY ESOPHAGEAL ADENOCARCINOMA

**DOI:** 10.1093/jcag/gwac036.158

**Published:** 2023-03-07

**Authors:** H Wilson, U Jogiat, A Bedard, P Blakely, W Sun, J Dang, S Karmali, E Bedard, C Wong

**Affiliations:** 1 University of Alberta; 2 Alberta Health Services, Edmonton, Canada

## Abstract

**Background:**

Previously, Barrett’s esophagus (BE) with high grade dysplasia (HGD) or neoplasia was treated with esophagectomy; however, recent guidelines support the use of endoscopic mucosal resection (EMR) for T1a esophageal adenocarcinoma (EAC) and potentially T1b EAC. Long term data for outcomes from EMR are lacking and these treatments are often provided with minimal collaboration between gastroenterologists and thoracic surgeons.

**Purpose:**

Our primary aim was to describe the findings from our multidisciplinary database of patients with Barrett’s esophagus and EAC undergoing endoscopic treatment. Secondary aims were to compare the overall survival and recurrence-free survival of patients undergoing endoscopic resection to those undergoing esophagectomy.

**Method:**

For the endoscopic resection cohort, a combined retrospective and prospective database was created containing demographic, clinical, and oncologic variables for patients undergoing endoscopic resection for early stage EAC from 2009 to 2021. For the esophagectomy cohort, a pre-existing retrospective database including patients undergoing esophagectomy for esophageal cancer from 2012 to 2018 at a single institution was used. A multivariate cox proportional hazards model was developed for recurrence-free survival and overall survival using a hypothesis driven approach. A kaplan-meier (KM) curve with associated log-rank test was created to evaluate recurrence-free survival and overall survival stratified by treatment modality.

**Result(s):**

A total of 108 patients were included in the analysis (73 EMR, 35 esophagectomy). Baseline characteristics including age, sex, and co-morbidities were similar among the two groups. KM curves stratified by treatment modality are provided in Figure 1. Esophagectomy was associated with greater DFS on univariate log-rank test (p = 0.0127), but no difference in OS (p = 0.9306). There was no significant difference between esophagectomy and endoscopic resection in the cox-model for OS (HR 1.03, 95% CI 0.45-2.32, p = 0.914). Endoscopic resection was associated with increased hazards of disease recurrence in the cox model for DFS (HR 2.56, 95% CI 1.1-6.0, p = 0.032). In the logistic regression model, high grade disease (OR 5.43, 95% CI 1.1 – 26.1, p = 0.035) and submucosal involvement (OR 7.8, 1.9-31.4, p = 0.004) were identified as significant predictors of positive margin necessitating esophagectomy after initial endoscopic resection. All patients who experienced a positive margin after endoscopic therapy were evaluated by a thoracic surgeon and proceeded to esophagectomy.

**Conclusion(s):**

In this largely retrospective analysis, our multidisciplinary approach was shown to be highly efficacious in the treatment of BE with EAC. Through optimizing collaboration between thoracic surgeons and gastroenterologists, patients receive the best therapeutic approach for their unique condition, taking into account oncologic factors and clinical comorbidities.

**Please acknowledge all funding agencies by checking the applicable boxes below:**

None

**Disclosure of Interest:**

None Declared

